# 5-Chloro-2-(4-fluoro­phen­yl)-3-phenyl­sulfinyl-1-benzofuran

**DOI:** 10.1107/S1600536811002686

**Published:** 2011-01-26

**Authors:** Hong Dae Choi, Pil Ja Seo, Byeng Wha Son, Uk Lee

**Affiliations:** a Department of Chemistry, Dongeui University, San 24 Kaya-dong Busanjin-gu, Busan 614-714, Republic of Korea; b Department of Chemistry, Pukyong National University, 599-1 Daeyeon 3-dong, Nam-gu, Busan 608-737, Republic of Korea

## Abstract

In the title compound, C_20_H_12_ClFO_2_S, the O atom and the phenyl ring of the phenyl­sulfinyl substituent lie on opposite sides of the plane of the benzofuran fragment; the phenyl ring is almost perpendicular to this plane [82.44 (5)°]. The 4-fluoro­phenyl ring is rotated out of the benzofuran plane, making a dihedral angle of 20.83 (6)°.

## Related literature

For the biological activity of benzofuran compounds, see: Aslam *et al.* (2006 ▶); Galal *et al.* (2009 ▶); Khan *et al.* (2005 ▶). For natural products with benzofuran rings, see: Akgul & Anil (2003 ▶); Soekamto *et al.* (2003 ▶). For our previous structural studies of related 5-halo-2-phenyl-3-phenyl­sulfinyl-1-benzofuran derivatives, see: Choi *et al.* (2009**a* ▶,*b* ▶,c* ▶).
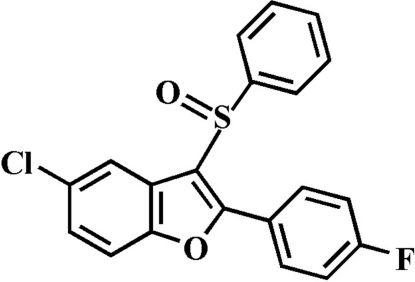


## Experimental

### 

#### Crystal data

C_20_H_12_ClFO_2_S
*M* *_r_* = 370.81
Triclinic, 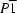

*a* = 8.2551 (2) Å
*b* = 9.4707 (3) Å
*c* = 11.4914 (3) Å
α = 71.403 (2)°
β = 81.707 (2)°
γ = 71.909 (2)°
*V* = 808.39 (4) Å^3^
*Z* = 2
Mo *K*α radiation
μ = 0.39 mm^−1^
*T* = 173 K
0.17 × 0.15 × 0.06 mm

#### Data collection

Bruker SMART APEXII CCD diffractometer
Absorption correction: multi-scan (*SADABS*; Bruker, 2009 ▶) *T*_min_ = 0.935, *T*_max_ = 0.978
14335 measured reflections
3722 independent reflections
2943 reflections with *I* > 2σ(*I*)
*R*_int_ = 0.031

#### Refinement

*R*[*F* ^2^ > 2σ(*F* ^2^)] = 0.036
*wR*(*F* ^2^) = 0.096
*S* = 1.04
3722 reflections
226 parameters
H-atom parameters constrained
Δρ_max_ = 0.37 e Å^−3^
Δρ_min_ = −0.35 e Å^−3^

### 

Data collection: *APEX2* (Bruker, 2009 ▶); cell refinement: *SAINT* (Bruker, 2009 ▶); data reduction: *SAINT*; program(s) used to solve structure: *SHELXS97* (Sheldrick, 2008 ▶); program(s) used to refine structure: *SHELXL97* (Sheldrick, 2008 ▶); molecular graphics: *ORTEP-3* (Farrugia, 1997 ▶) and *DIAMOND* (Brandenburg, 1998 ▶); software used to prepare material for publication: *SHELXL97*.

## Supplementary Material

Crystal structure: contains datablocks global, I. DOI: 10.1107/S1600536811002686/rn2082sup1.cif

Structure factors: contains datablocks I. DOI: 10.1107/S1600536811002686/rn2082Isup2.hkl

Additional supplementary materials:  crystallographic information; 3D view; checkCIF report

## Figures and Tables

**Fig. 1.**
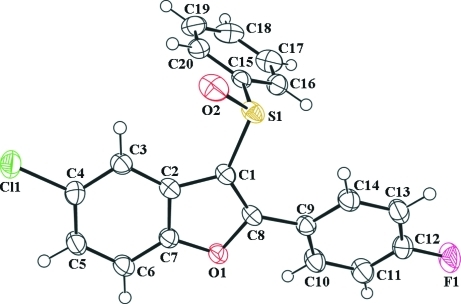
The molecular structure of the title compound with the atom numbering scheme. Displacement ellipsoids are drawn at the 50% probability level. H atoms are presented as a small spheres of arbitrary radius.

## supplementary crystallographic information

### Comment

Many compounds having a benzofuran ring system have attracted much attention owing to their pharmacological properties such as antifungal, antimicrobial, antitumor and antiviral activities (Aslam *et al.*, 2006; Galal *et al.*, 2009; Khan *et al.*, 2005). These compounds occur in a wide range of natural products (Akgul & Anil, 2003; Soekamto *et al.*, 2003). As part of our ongoing program of the substituent effect on the solid state structures of 5-halo-2-phenyl-3-phenylsulfinyl-1-benzofuran analogues (Choi *et al.*, 2009*a*,*b*,*c*), we report herein on the crystal structure of the title compound.

In the title molecule (Fig. 1), the benzofuran unit is essentially planar, with a mean deviation of 0.011 (1) Å from the least-squares plane defined by the nine constituent atoms. The phenyl ring makes a dihedral angle of 82.44 (5)° with the mean plane of the benzofuran fragment. The dihedral angle formed by the mean plane of the benzofuran fragment and the 4-fluorophenyl ring is 20.83 (6)°.

### Experimental

77% 3-chloroperoxybenzoic acid (179 mg, 0.8 mmol) was added in small portions to a stirred solution of 5-chloro-2-(4-fluorophenyl)-3-phenylsulfanyl-1-benzofuran (284 mg, 0.8 mmol) in dichloromethane (30 mL) at 273 K. After being stirred at room temperature for 4h, the mixture was washed with saturated sodium bicarbonate solution and the organic layer was separated, dried over magnesium sulfate, filtered and concentrated at reduced pressure. The residue was purified by column chromatography (hexane–ethyl ace tate, 2:1 v/v) to afford the title compound as a colorless solid [yield 76%, m.p. 480-481 K; *R*_f_ = 0.68 (hexane–ethyl acetate, 2:1 v/v)]. Single crystals suitable for X-ray diffraction were prepared by slow evaporation of a solution of the title compound in benzene at room temperature.

### Refinement

All H atoms were positioned geometrically and refined using a riding model, with C—H = 0.95 Å for aryl H atoms. *U*_iso_(H) = 1.2*U*_eq_(C) for aryl H atoms.

### Crystal data

**Table:**

C_20_H_12_ClFO_2_S	*Z* = 2
*M**_r_* = 370.81	*F*(000) = 380
Triclinic, *P*1	*D*_x_ = 1.523 Mg m^−^^3^
Hall symbol: -P 1	Mo *K*α radiation, λ = 0.71073 Å
*a* = 8.2551 (2) Å	Cell parameters from 5406 reflections
*b* = 9.4707 (3) Å	θ = 2.6–27.3°
*c* = 11.4914 (3) Å	µ = 0.39 mm^−^^1^
α = 71.403 (2)°	*T* = 173 K
β = 81.707 (2)°	Block, colourless
γ = 71.909 (2)°	0.17 × 0.15 × 0.06 mm
*V* = 808.39 (4) Å^3^	

### Data collection

**Table:**

Bruker SMART APEXII CCD diffractometer	3722 independent reflections
Radiation source: rotating anode	2943 reflections with *I* > 2σ(*I*)
graphite multilayer	*R*_int_ = 0.031
Detector resolution: 10.0 pixels mm^-1^	θ_max_ = 27.7°, θ_min_ = 1.9°
φ and ω scans	*h* = −10→10
Absorption correction: multi-scan (*SADABS*; Bruker, 2009)	*k* = −12→12
*T*_min_ = 0.935, *T*_max_ = 0.978	*l* = −15→14
14335 measured reflections	

### Refinement

**Table:**

Refinement on *F*^2^	Primary atom site location: structure-invariant direct methods
Least-squares matrix: full	Secondary atom site location: difference Fourier map
*R*[*F*^2^ > 2σ(*F*^2^)] = 0.036	Hydrogen site location: difference Fourier map
*wR*(*F*^2^) = 0.096	H-atom parameters constrained
*S* = 1.04	*w* = 1/[σ^2^(*F*_o_^2^) + (0.0461*P*)^2^ + 0.1974*P*] where *P* = (*F*_o_^2^ + 2*F*_c_^2^)/3
3722 reflections	(Δ/σ)_max_ = 0.001
226 parameters	Δρ_max_ = 0.37 e Å^−^^3^
0 restraints	Δρ_min_ = −0.35 e Å^−^^3^

### Special details

**Table:**

Geometry. All esds (except the esd in the dihedral angle between two l.s. planes) are estimated using the full covariance matrix. The cell esds are taken into account individually in the estimation of esds in distances, angles and torsion angles; correlations between esds in cell parameters are only used when they are defined by crystal symmetry. An approximate (isotropic) treatment of cell esds is used for estimating esds involving l.s. planes.
Refinement. Refinement of F^2^ against ALL reflections. The weighted R-factor wR and goodness of fit S are based on F^2^, conventional R-factors R are based on F, with F set to zero for negative F^2^. The threshold expression of F^2^ > 2sigma(F^2^) is used only for calculating R-factors(gt) etc. and is not relevant to the choice of reflections for refinement. R-factors based on F^2^ are statistically about twice as large as those based on F, and R- factors based on ALL data will be even larger.

### Fractional atomic coordinates and isotropic or equivalent isotropic displacement parameters (Å^2^)

**Table:**

	*x*	*y*	*z*	*U*_iso_*/*U*_eq_	
Cl1	1.03501 (6)	0.73080 (5)	0.38102 (4)	0.04197 (14)	
S1	0.62029 (5)	0.60069 (5)	0.86187 (4)	0.03045 (12)	
F1	0.03640 (16)	1.10463 (14)	1.14539 (11)	0.0571 (3)	
O1	0.52873 (15)	1.05149 (12)	0.68603 (10)	0.0308 (3)	
O2	0.79096 (15)	0.50153 (14)	0.83462 (13)	0.0435 (3)	
C1	0.6061 (2)	0.79137 (18)	0.76450 (15)	0.0268 (3)	
C2	0.6984 (2)	0.82981 (18)	0.64745 (15)	0.0273 (3)	
C3	0.8176 (2)	0.74611 (19)	0.57773 (15)	0.0293 (4)	
H3	0.8542	0.6358	0.6037	0.035*	
C4	0.8803 (2)	0.8308 (2)	0.46888 (16)	0.0314 (4)	
C5	0.8269 (2)	0.9928 (2)	0.42678 (16)	0.0352 (4)	
H5	0.8734	1.0459	0.3510	0.042*	
C6	0.7069 (2)	1.0758 (2)	0.49497 (16)	0.0338 (4)	
H6	0.6679	1.1860	0.4679	0.041*	
C7	0.6461 (2)	0.99115 (18)	0.60439 (15)	0.0279 (3)	
C8	0.5071 (2)	0.92768 (18)	0.78381 (15)	0.0278 (3)	
C9	0.3831 (2)	0.97013 (19)	0.88009 (15)	0.0283 (4)	
C10	0.2638 (2)	1.1155 (2)	0.85298 (17)	0.0378 (4)	
H10	0.2632	1.1842	0.7720	0.045*	
C11	0.1463 (2)	1.1617 (2)	0.94148 (18)	0.0434 (5)	
H11	0.0651	1.2611	0.9227	0.052*	
C12	0.1501 (2)	1.0603 (2)	1.05693 (17)	0.0388 (4)	
C13	0.2645 (2)	0.9155 (2)	1.08844 (17)	0.0391 (4)	
H13	0.2634	0.8479	1.1697	0.047*	
C14	0.3814 (2)	0.8707 (2)	0.99897 (16)	0.0347 (4)	
H14	0.4616	0.7708	1.0188	0.042*	
C15	0.4679 (2)	0.56305 (17)	0.78808 (15)	0.0274 (3)	
C16	0.2970 (2)	0.6120 (2)	0.82334 (17)	0.0340 (4)	
H16	0.2621	0.6660	0.8837	0.041*	
C17	0.1781 (2)	0.5810 (2)	0.7692 (2)	0.0449 (5)	
H17	0.0602	0.6143	0.7919	0.054*	
C18	0.2304 (3)	0.5017 (2)	0.6824 (2)	0.0478 (5)	
H18	0.1481	0.4809	0.6452	0.057*	
C19	0.4007 (3)	0.4522 (2)	0.64904 (19)	0.0470 (5)	
H19	0.4355	0.3970	0.5895	0.056*	
C20	0.5211 (2)	0.48253 (19)	0.70198 (17)	0.0371 (4)	
H20	0.6389	0.4485	0.6794	0.045*	

### Atomic displacement parameters (Å^2^)

**Table:**

	*U*^11^	*U*^22^	*U*^33^	*U*^12^	*U*^13^	*U*^23^
Cl1	0.0389 (3)	0.0426 (3)	0.0377 (3)	−0.0065 (2)	0.01078 (19)	−0.0129 (2)
S1	0.0269 (2)	0.0251 (2)	0.0310 (2)	−0.00523 (17)	−0.00234 (17)	0.00144 (17)
F1	0.0572 (8)	0.0530 (7)	0.0476 (7)	−0.0058 (6)	0.0243 (6)	−0.0174 (6)
O1	0.0347 (6)	0.0239 (6)	0.0285 (6)	−0.0075 (5)	0.0045 (5)	−0.0038 (5)
O2	0.0259 (6)	0.0327 (7)	0.0566 (9)	0.0006 (5)	−0.0028 (6)	−0.0010 (6)
C1	0.0249 (8)	0.0252 (8)	0.0264 (8)	−0.0072 (6)	−0.0010 (6)	−0.0022 (6)
C2	0.0248 (8)	0.0263 (8)	0.0282 (8)	−0.0087 (7)	−0.0020 (6)	−0.0027 (7)
C3	0.0259 (8)	0.0259 (8)	0.0325 (9)	−0.0060 (7)	−0.0008 (7)	−0.0053 (7)
C4	0.0275 (8)	0.0349 (9)	0.0301 (9)	−0.0072 (7)	0.0023 (7)	−0.0101 (7)
C5	0.0367 (9)	0.0359 (9)	0.0288 (9)	−0.0132 (8)	0.0055 (7)	−0.0040 (7)
C6	0.0378 (10)	0.0268 (8)	0.0314 (9)	−0.0097 (7)	0.0020 (7)	−0.0023 (7)
C7	0.0281 (8)	0.0253 (8)	0.0279 (8)	−0.0072 (7)	0.0006 (7)	−0.0061 (7)
C8	0.0274 (8)	0.0262 (8)	0.0261 (8)	−0.0096 (7)	−0.0009 (7)	−0.0010 (6)
C9	0.0263 (8)	0.0285 (8)	0.0296 (9)	−0.0094 (7)	0.0009 (7)	−0.0074 (7)
C10	0.0354 (9)	0.0332 (9)	0.0343 (10)	−0.0046 (8)	0.0026 (8)	−0.0025 (8)
C11	0.0384 (10)	0.0351 (10)	0.0450 (11)	−0.0021 (8)	0.0072 (9)	−0.0078 (9)
C12	0.0358 (10)	0.0411 (10)	0.0389 (10)	−0.0118 (8)	0.0117 (8)	−0.0160 (8)
C13	0.0444 (11)	0.0379 (10)	0.0296 (9)	−0.0132 (8)	0.0057 (8)	−0.0045 (8)
C14	0.0364 (9)	0.0284 (9)	0.0329 (9)	−0.0055 (7)	0.0013 (7)	−0.0051 (7)
C15	0.0277 (8)	0.0194 (7)	0.0286 (8)	−0.0054 (6)	0.0018 (7)	−0.0008 (6)
C16	0.0289 (9)	0.0309 (9)	0.0382 (10)	−0.0064 (7)	0.0030 (7)	−0.0089 (8)
C17	0.0298 (9)	0.0388 (11)	0.0615 (13)	−0.0084 (8)	−0.0043 (9)	−0.0089 (10)
C18	0.0532 (13)	0.0326 (10)	0.0604 (13)	−0.0146 (9)	−0.0191 (11)	−0.0078 (9)
C19	0.0673 (14)	0.0312 (10)	0.0445 (11)	−0.0142 (10)	−0.0020 (10)	−0.0136 (9)
C20	0.0381 (10)	0.0257 (9)	0.0415 (10)	−0.0064 (8)	0.0075 (8)	−0.0084 (8)

### Geometric parameters (Å, °)

**Table:**

Cl1—C4	1.7422 (17)	C9—C10	1.391 (2)
S1—O2	1.4845 (13)	C10—C11	1.379 (3)
S1—C1	1.7732 (16)	C10—H10	0.9500
S1—C15	1.7911 (17)	C11—C12	1.365 (3)
F1—C12	1.358 (2)	C11—H11	0.9500
O1—C7	1.3717 (19)	C12—C13	1.371 (3)
O1—C8	1.3788 (19)	C13—C14	1.381 (2)
C1—C8	1.363 (2)	C13—H13	0.9500
C1—C2	1.445 (2)	C14—H14	0.9500
C2—C3	1.390 (2)	C15—C20	1.379 (2)
C2—C7	1.391 (2)	C15—C16	1.385 (2)
C3—C4	1.381 (2)	C16—C17	1.381 (3)
C3—H3	0.9500	C16—H16	0.9500
C4—C5	1.396 (2)	C17—C18	1.379 (3)
C5—C6	1.378 (2)	C17—H17	0.9500
C5—H5	0.9500	C18—C19	1.376 (3)
C6—C7	1.379 (2)	C18—H18	0.9500
C6—H6	0.9500	C19—C20	1.380 (3)
C8—C9	1.461 (2)	C19—H19	0.9500
C9—C14	1.391 (2)	C20—H20	0.9500
			
O2—S1—C1	106.64 (7)	C11—C10—H10	119.4
O2—S1—C15	107.22 (8)	C9—C10—H10	119.4
C1—S1—C15	96.93 (7)	C12—C11—C10	118.01 (17)
C7—O1—C8	106.94 (12)	C12—C11—H11	121.0
C8—C1—C2	107.22 (14)	C10—C11—H11	121.0
C8—C1—S1	127.42 (13)	F1—C12—C11	118.74 (17)
C2—C1—S1	125.36 (12)	F1—C12—C13	118.13 (17)
C3—C2—C7	119.50 (15)	C11—C12—C13	123.13 (17)
C3—C2—C1	135.44 (15)	C12—C13—C14	118.27 (17)
C7—C2—C1	105.05 (14)	C12—C13—H13	120.9
C4—C3—C2	116.85 (15)	C14—C13—H13	120.9
C4—C3—H3	121.6	C13—C14—C9	120.76 (16)
C2—C3—H3	121.6	C13—C14—H14	119.6
C3—C4—C5	123.08 (16)	C9—C14—H14	119.6
C3—C4—Cl1	118.37 (13)	C20—C15—C16	121.39 (17)
C5—C4—Cl1	118.54 (13)	C20—C15—S1	120.46 (13)
C6—C5—C4	120.11 (16)	C16—C15—S1	118.11 (13)
C6—C5—H5	119.9	C17—C16—C15	118.85 (17)
C4—C5—H5	119.9	C17—C16—H16	120.6
C5—C6—C7	116.72 (15)	C15—C16—H16	120.6
C5—C6—H6	121.6	C18—C17—C16	120.02 (19)
C7—C6—H6	121.6	C18—C17—H17	120.0
O1—C7—C6	125.76 (14)	C16—C17—H17	120.0
O1—C7—C2	110.51 (14)	C19—C18—C17	120.58 (19)
C6—C7—C2	123.72 (16)	C19—C18—H18	119.7
C1—C8—O1	110.27 (14)	C17—C18—H18	119.7
C1—C8—C9	135.02 (15)	C18—C19—C20	120.14 (19)
O1—C8—C9	114.67 (14)	C18—C19—H19	119.9
C14—C9—C10	118.60 (16)	C20—C19—H19	119.9
C14—C9—C8	122.21 (15)	C15—C20—C19	119.01 (18)
C10—C9—C8	119.19 (15)	C15—C20—H20	120.5
C11—C10—C9	121.23 (17)	C19—C20—H20	120.5
			
O2—S1—C1—C8	−154.59 (15)	C7—O1—C8—C9	−179.06 (13)
C15—S1—C1—C8	95.06 (16)	C1—C8—C9—C14	22.4 (3)
O2—S1—C1—C2	26.14 (16)	O1—C8—C9—C14	−160.06 (15)
C15—S1—C1—C2	−84.21 (15)	C1—C8—C9—C10	−158.49 (19)
C8—C1—C2—C3	179.31 (18)	O1—C8—C9—C10	19.1 (2)
S1—C1—C2—C3	−1.3 (3)	C14—C9—C10—C11	0.4 (3)
C8—C1—C2—C7	0.04 (18)	C8—C9—C10—C11	−178.78 (17)
S1—C1—C2—C7	179.44 (12)	C9—C10—C11—C12	−0.1 (3)
C7—C2—C3—C4	1.3 (2)	C10—C11—C12—F1	179.39 (17)
C1—C2—C3—C4	−177.90 (17)	C10—C11—C12—C13	−0.1 (3)
C2—C3—C4—C5	−1.2 (3)	F1—C12—C13—C14	−179.41 (16)
C2—C3—C4—Cl1	177.97 (12)	C11—C12—C13—C14	0.1 (3)
C3—C4—C5—C6	0.3 (3)	C12—C13—C14—C9	0.1 (3)
Cl1—C4—C5—C6	−178.87 (14)	C10—C9—C14—C13	−0.4 (3)
C4—C5—C6—C7	0.5 (3)	C8—C9—C14—C13	178.74 (16)
C8—O1—C7—C6	−178.57 (16)	O2—S1—C15—C20	−13.01 (16)
C8—O1—C7—C2	0.94 (18)	C1—S1—C15—C20	96.85 (14)
C5—C6—C7—O1	179.02 (16)	O2—S1—C15—C16	164.67 (12)
C5—C6—C7—C2	−0.4 (3)	C1—S1—C15—C16	−85.46 (14)
C3—C2—C7—O1	179.98 (14)	C20—C15—C16—C17	−1.0 (2)
C1—C2—C7—O1	−0.61 (18)	S1—C15—C16—C17	−178.63 (14)
C3—C2—C7—C6	−0.5 (3)	C15—C16—C17—C18	0.4 (3)
C1—C2—C7—C6	178.91 (16)	C16—C17—C18—C19	0.3 (3)
C2—C1—C8—O1	0.54 (18)	C17—C18—C19—C20	−0.4 (3)
S1—C1—C8—O1	−178.84 (11)	C16—C15—C20—C19	0.8 (3)
C2—C1—C8—C9	178.16 (17)	S1—C15—C20—C19	178.43 (14)
S1—C1—C8—C9	−1.2 (3)	C18—C19—C20—C15	−0.1 (3)
C7—O1—C8—C1	−0.91 (18)		
